# Altered Dynamic Amplitude of Low-Frequency Fluctuations in Patients With Migraine Without Aura

**DOI:** 10.3389/fnhum.2021.636472

**Published:** 2021-02-10

**Authors:** Hong Chen, Guiqiang Qi, Yingxia Zhang, Ying Huang, Shaojin Zhang, Dongjun Yang, Junwei He, Lan Mu, Lin Zhou, Min Zeng

**Affiliations:** Department of Radiology, The Third Affiliated Hospital of Chengdu Medical College, Pidu District People's Hospital, Chengdu, China

**Keywords:** migraine without aura, dynamic amplitude of low-frequency fluctuations, resting-state, functional MRI, classification 3

## Abstract

Migraine is a chronic and idiopathic disorder leading to cognitive and affective problems. However, the neural basis of migraine without aura is still unclear. In this study, dynamic amplitude of low-frequency fluctuations (dALFF) analyses were performed in 21 patients with migraine without aura and 21 gender- and age-matched healthy controls to identify the voxel-level abnormal functional dynamics. Significantly decreased dALFF in the bilateral anterior insula, bilateral lateral orbitofrontal cortex, bilateral medial prefrontal cortex, bilateral anterior cingulate cortex, and left middle frontal cortex were found in patients with migraine without aura. The dALFF values in the anterior cingulate cortex were negatively correlated with pain intensity, i.e., visual analog scale. Finally, support vector machine was used to classify patients with migraine without aura from healthy controls and achieved an accuracy of 83.33%, sensitivity of 90.48%, and specificity of 76.19%. Our findings provide the evidence that migraine influences the brain functional activity dynamics and reveal the neural basis for migraine, which could facilitate understanding the neuropathology of migraine and future treatment.

## Introduction

Migraine is an idiopathic and chronic disorder influencing the life quality of patients (Kruit et al., 2004). The frequent migraine attacks also affect patients' mental and physical health (Tietjen, 2004; Borsook et al., 2012). The previous studies have demonstrated that long-term chronic pain leads to functional damages in sensory, cognition, and affective processing (Montero-Homs, 2009; Linton, 2013; Denkinger et al., 2014). A lot of previous studies have revealed that migraine induced brain gray matter volume and white matter integrity changes using structural and diffusion magnetic resonance imaging (MRI) (DaSilva et al., 2007; Kim et al., 2008; Schmidt-Wilcke et al., 2008; Valfrè et al., 2008). With the use of resting-state functional MRI (fMRI), altered local regional homogeneity and whole-brain functional connectivity homogeneity were found in patients with migraine (Yu et al., 2012; Zhang et al., 2020). All these findings suggest that migraine could change the brain structure and functions.

The low-frequency oscillation of the brain is mainly characterized using resting-state fMRI with blood oxygen level-dependent (BOLD) signals (Biswal et al., 1995; Fox and Raichle, 2007). Resting-state fMRI has been widely applied to study the brain functional organization (Greicius et al., 2003; Fox et al., 2006; Wang et al., 2015, 2017c; Xu et al., 2019; Gao et al., 2020), to identify the functional abnormalities in patients with brain disorders (Greicius et al., 2007; Brier et al., 2012; Muller et al., 2013; Wu et al., 2016; Sun et al., 2018), and to reveal the neural basis of treatment response (Guo et al., 2012; Mulders et al., 2016; Wang et al., 2017b, 2018; Xu et al., 2020). To quantitatively measure low-frequency oscillation, the amplitude of low-frequency fluctuations (ALFF) is proposed (Zang et al., 2007). Using ALFF, Xue et al. (2013) found decreased ALFF values in the left rostral anterior cingulate cortex and bilateral prefrontal cortex as well as increased ALFF values in the right thalamus. Using ALFF to reveal the early abnormal functional activity is promising to explore early marker for migraine. Recently, using sliding-window approach, the dynamic ALFF (dALFF) method was developed by calculating the variance of ALFF over time (Fu et al., 2018). The dALFF has been applied to study the functional activity abnormalities in subjects under disease state and provides some new evidence (Fu et al., 2018; Ma et al., 2020; Pang et al., 2020). Thus, to reveal the dynamic changes of the low-frequency oscillation in migraine patients may provide supplementary information to understand its neural basis.

In this study, with 21 patients with migraine without aura and 21 gender- and age-matched healthy controls, a voxel-wise dALFF method was used to reveal the dynamic changes of low-frequency oscillation in migraine patients. Moreover, using the dALFF values in brain regions showing differences between patients and controls as features, support vector machine (SVM) was used to classify migraine patients from healthy controls to further validate the results.

## Materials and Methods

### Participants

In this study, we recruited 21 right-handed patients with migraine without aura (female/male = 16/5; age = 31.19 ± 6.38 years) and 21 gender- and age-matched right-handed healthy controls (female/male = 13/8; age = 30.19 ± 6.3 years) at the Third Affiliated Hospital of Chengdu Medical College, Pidu District People's Hospital. The diagnosis of migraine without aura was based on the International Headache Society criteria. The inclusion criteria for patients with migraine without aura were follows: (1) no migraine precipitated during or on the day after the scan; (2) did not suffer from a migraine attack at least 72 h before the experiment; (3) for migraine patients and healthy controls, no lifetime history of seizures, head trauma, serious medical or surgical illness, substance dependence or abuse, and contraindications for MRI. The participants were excluded if structural abnormalities were detected on MRI examination, and no subject with structural deficits was found. Written informed consent was provided and obtained from all the subjects. This study was approved by the local ethics committees of the Third Affiliated Hospital of Chengdu Medical College, Pidu District People's Hospital. The detailed information for the subjects can be found in our previous study (Zhang et al., 2020).

### Resting-State Functional MRI Data Acquisition

MRI data were acquired on a 3-Tesla Siemens MRI scanner in the Department of Radiology, the Third Affiliated Hospital of Chengdu Medical College, Pidu District People's Hospital of Chengdu, China. The participants were instructed to close their eyes and not fall asleep, and earplugs and foam padding were used to reduce scanner noise and head motion. Resting-state fMRI data were acquired using a gradient-echo echo-planar imaging (GRE-EPI) sequence with the following parameters: repetition time (TR) = 2,000 ms, echo time (TE) = 30 ms, flip angle (FA) = 90°, matrix = 64 × 64, field of view (FOV) = 220 × 220 mm, slice thickness = 4 mm with inter-slice gap = 0.6 mm, 32 axial slices, and 250 time points. The information can be found in our previous study (Zhang et al., 2020).

### Resting-State Functional MRI Data Preprocessing

The resting-state fMRI data were preprocessed using the toolkit of DPARSF version 2.3 (Chao-Gan and Yu-Feng, 2010) (www.restfmri.net/forum/DPARSF). To exclude unstable magnetization effect, the first 10 volumes were discarded. Then, all the remaining volumes were realigned to the first volume to correct head motion. Next, all the images were normalized to standard EPI template in Montreal Neurological Institute (MNI) space and resampled to 3-mm voxel resolution. Subsequently, 6-mm Gaussian kernel was used to smooth the fMRI data; and Friston 24-parameter of head motion, white matter, cerebrospinal fluid, and global mean signals were regressed out. Finally, the resting-state data were filtered with a frequency band of 0.01–0.08 Hz for dALFF analyses. To remove head-motion effects, the subjects were excluded if the head movement exceeds 3 mm or 3°. Additionally, “scrubbing” was used to delete the bad images before 2 time points and after 1 time point exceeding the preset criteria [frame displacement (FD) <0.5] (Power et al., 2012). We also calculated the mean FD values of healthy controls and migraine patients, and the two-sample *t*-test was used and did not find the significant difference (FD values of patients = 0.16 ± 0.29; FD values of controls = 0.15 ± 0.027; *p* = 0.49).

### Dynamic Amplitude of Low-Frequency Fluctuations Calculation

The ALFF was proposed to characterize the resting-state functional activity of each voxel in brain (Zou et al., 2008). To calculate ALFF, the time series was first transformed to frequency domain, and the ALFF is computed at the power within the low-frequency range of 0.01–0.8 Hz. To calculate dALFF, a sliding window method was used. The length of sliding window is determined based on the criterion that minimum window length should be larger than 1/*f*_min_, where *f*_min_ is the minimum frequency of time series (Leonardi and Van De Ville, 2015; Du et al., 2017; Li et al., 2019). Finally, a window length of 50 TR with step size of 5 TR was applied in this study. The ALFF map was computed in each window, and the variance of the ALFF maps across all the windows was computed to measure the dynamic. The dALFF maps were transformed to z-scores for statistical analyses. Two-sample *t*-test was performed to compare the dALFF maps between healthy controls and patients with migraine without aura. The significant level was set at *p* < 0.05 using a Gaussian random field (GRF) correction method. To further validate the results obtained with the window length of 50 TR, the window length of 30 and 70 TR were further applied.

### Correlation Analyses

To explore the relationship between dALFF and clinical measures, correlation analyses were performed between the dALFF values of the areas showing differences in patients and visual analog scale (VAS), and disease duration. The significance was set at *p* < 0.05 with Bonferroni correction.

### Support Vector Machine Classification

A linear SVM classifier was performed using LIBSVM software (Chang and Lin, 2011). For classification, the mean dALFF values of the areas showing differences between patients and controls were used as the features. To estimate the performance of our classifier, a leave-one-out cross-validation (LOOCV) test was used to assess the generalization ability because of our limited number of samples in the present study. The classification result was assessed using the classification accuracy, sensitivity, and specificity.

## Results

### Clinical Characteristics

There were no significant differences in age (*p* = 0.61) and sex (*p* = 0.51) between healthy controls and patients with migraine without aura (Table 1).

**Table 1:** Demographics and clinical characteristics of the subjects used in present study.

	**Migraine (*n* = 21)**	**Controls (*n* = 21)**	***p***
Gender (male/female)	16/5	13/8	0.51
Age (mean ± SD)	31.19 ± 6.38	30.19 ± 6.3	0.61
HAMA (mean ± SD)	7.95 ± 6.77		
HAMD (mean ± SD)	6.71 ± 6.25		
Duration of illness (months)	44.69 ± 61.13		
VAS (mean ± SD)	4.33 ± 1.46		

*A Pearson chi-squared test was used for gender comparison. Two-sample t-tests were used for age comparisons*.

*VAS, visual analog scale; HAMD, Hamilton Depression Rating Scale; HAMA, Hamilton Anxiety Scale*.

### Changed Dynamic Amplitude of Low-Frequency Fluctuations

**Figure 1:**
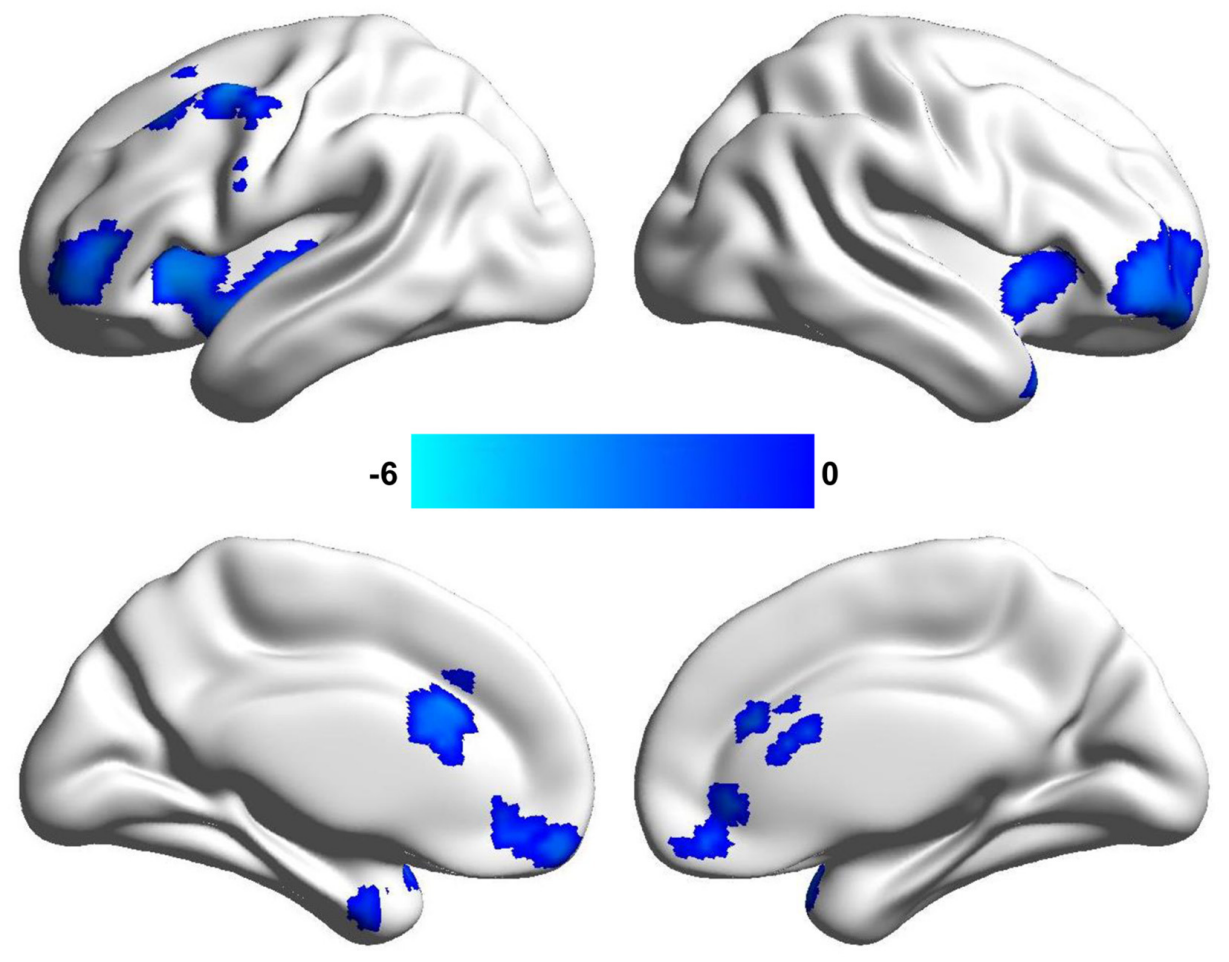
Changed dynamic amplitude of low-frequency fluctuations (ALFF) in window length of 50 repetition time (TR) in patients with migraine without aura. Significantly decreased dynamic ALFF in the bilateral anterior insula, bilateral anterior cingulate cortex, bilateral lateral orbitofrontal cortex, and left middle frontal cortex were found in patients with migraine without aura.

**Figure 2:**
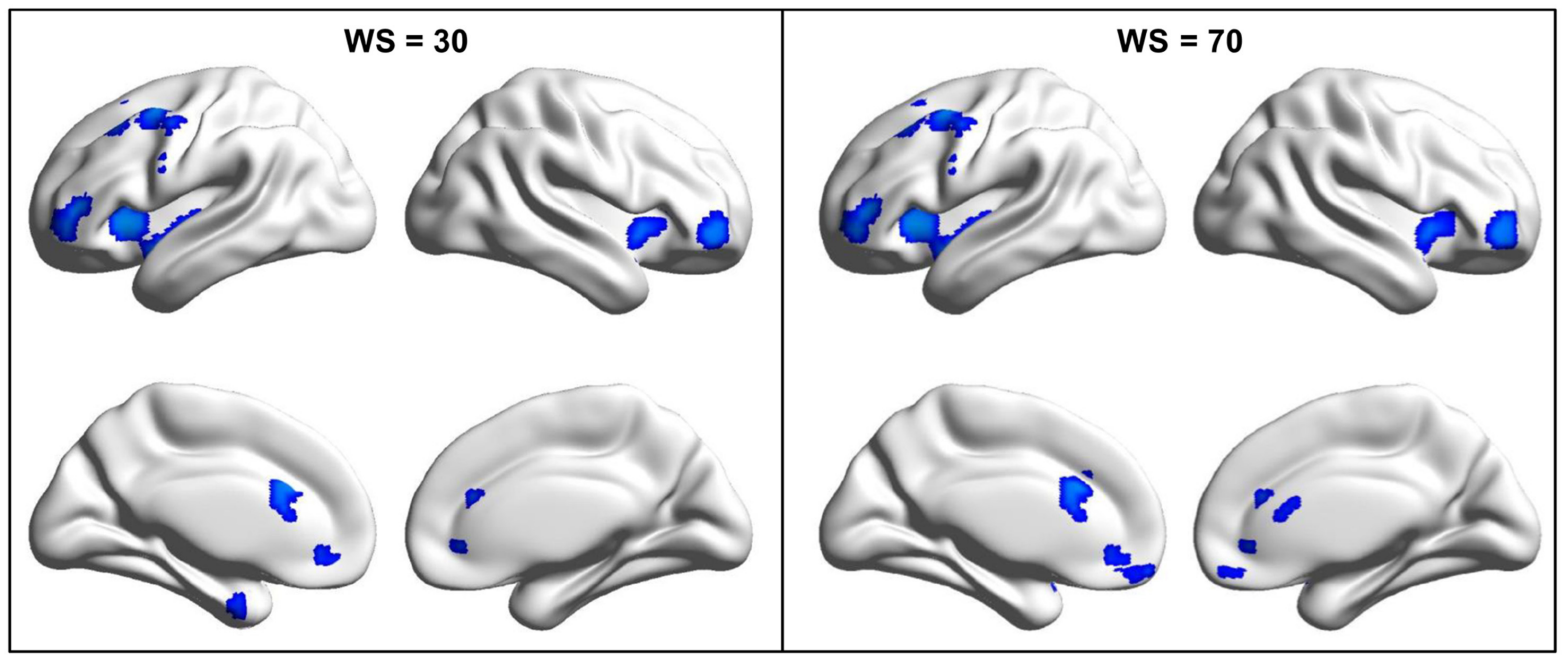
Changed dynamic amplitude of low-frequency fluctuations (ALFF) in migraine patients was validated in window length of 30 and 70 repetition times (TRs). To validate the findings obtained with window length of 50 TR, the same procedures were performed in window length of 30 and 70 TRs and found similar results.

By the analysis of dALFF in window length of 50 TR, significantly decreased dALFF in the bilateral anterior insula, bilateral lateral orbitofrontal cortex, bilateral medial prefrontal cortex, bilateral anterior cingulate cortex, and left middle frontal cortex in patients with migraine without aura compared with healthy controls (Figure 1 and Table 2) was found. We also analyzed dALFF in window length of 30 and 70 TR, and similar results were found (Figure 2).

**Table 2:** Regions with decreased dynamical amplitude of low-frequency fluctuations in patients with migraine without aura vs. healthy controls.

**Brain regions**	**Peak MNI coordinates**			**t values**	**Cluster size**
	**X**	**Y**	**Z**		
Anterior insula	−39	18	0	−4.71	383
Anterior insula	42	15	−9	−4.41	136
Lateral orbitofrontal cortex	−39	39	6	−4.53	185
Lateral orbitofrontal cortex	42	45	−3	−3.89	266
Middle frontal cortex	−46	0	51	−4.55	301
Medial prefrontal cortex	−3	54	−15	−3.74	118
Anterior cingulate cortex	−6	24	27	−3.87	102

### Correlation Analyses

**Figure 3:**
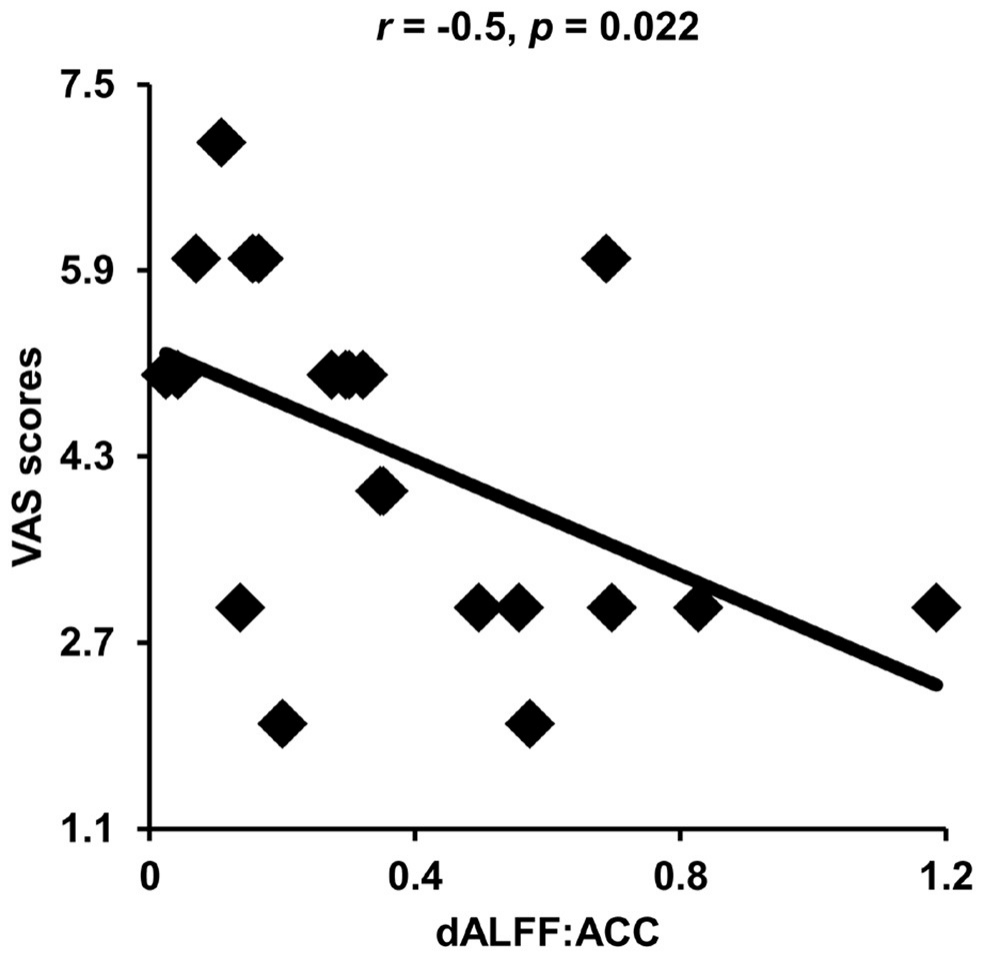
Correlation analyses between dynamic amplitude of low-frequency fluctuations (ALFF) and clinical measures. Significant correlation was found between the dynamic ALFF in the anterior cingulate cortex and visual analog scale (VAS) scores in migraine patients.

Correlation analyses identified significantly negative correlations between dALFF values in the anterior cingulate cortex and VAS scores (*r* = −0.5, *p* = 0.022) in patients with migraine without aura after multiple comparisons correction (Figure 3).

### Classification Results

**Figure 4:**
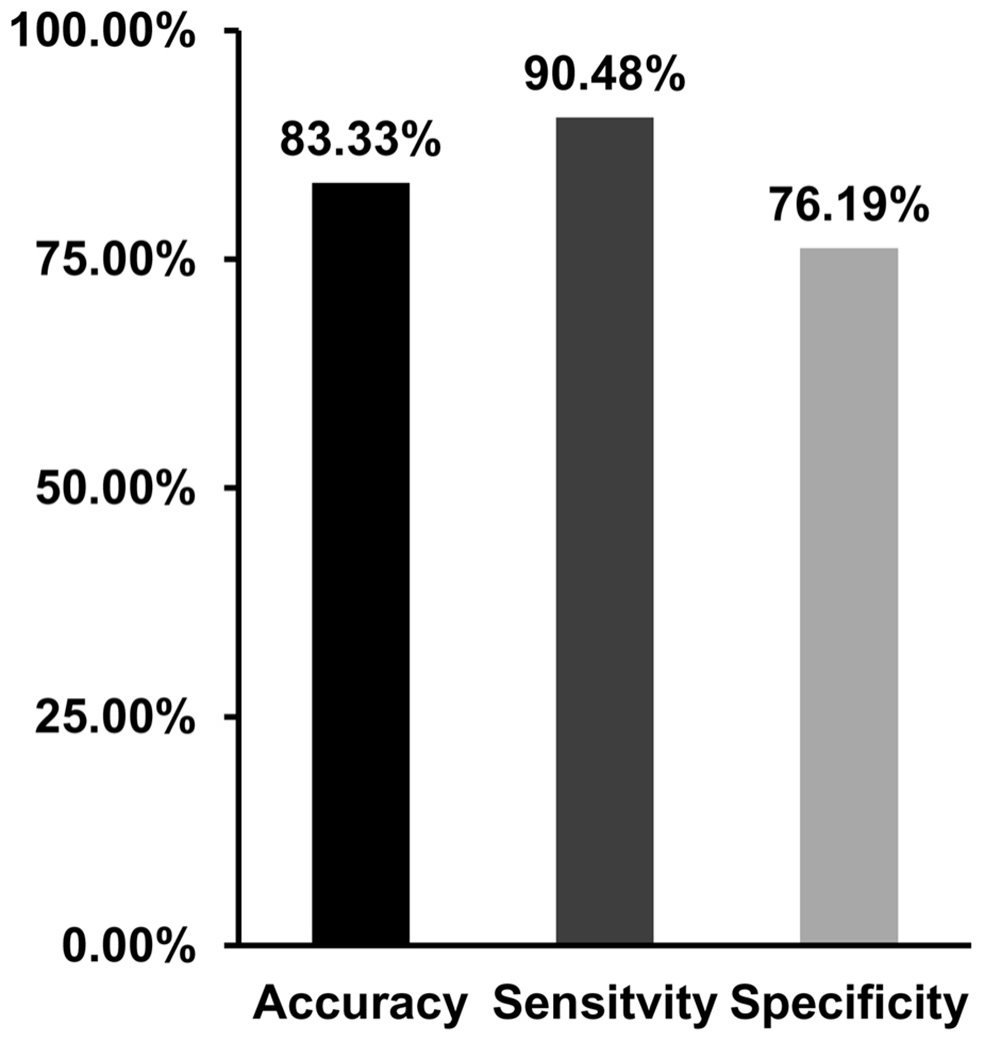
Support vector machine (SVM) was applied to classify migraine patients and healthy controls with the dynamic amplitude of low-frequency fluctuations (ALFF) values in brain areas showing differences between patients and controls as features. SVM achieves a classification accuracy of 83.33%, sensitivity of 90.48%, and specificity of 76.19%.

With the use of the changed dALFF values as features, experimental results showed a correct classification rate of 83.33%, sensitivity of 90.4%, and specificity of 76.19% using a leave-one-out cross-validation method (Figure 4).

## Discussion

In our study, dALFF method was applied and identified decreased dALFF in the bilateral anterior insula, bilateral orbitofrontal cortex, bilateral anterior cingulate cortex, bilateral medial prefrontal cortex, and left middle frontal cortex; and the changed dALFF in the anterior cingulate cortex was negatively correlated with VAS scores. Moreover, the changed dALFF can serve as an effective neuromarker to differentiate patients with migraine without aura from controls. Our findings revealed dynamic changes of brain functional activities in migraine patients and provide new evidence of neurophysiological abnormalities in migraine.

We found decreased dALFF in the anterior cingulate cortex and anterior insula in migraine patients without aura. The anterior insula and anterior cingulate cortex are two important nodes of salience network, which is mainly involved in coordinating the dynamic interaction between the external orient stimulus and internal self-perception (Menon and Uddin, 2010; Menon, 2011; Wang et al., 2017a, 2019b). The functional abnormality of anterior cingulate cortex and anterior insula found in our study is supported by previous functional and structural studies (Peyron et al., 2000; Kim et al., 2008; Valfrè et al., 2008). Importantly, we found that decreased dALFF in the anterior cingulate cortex is associated with clinical pain intensity. Thus, the abnormal dynamic functional activities in the anterior cingulate cortex may be a neuromarker to predict the pain intensity.

The decreased dALFF in the medial prefrontal cortex, middle frontal cortex, and lateral orbitofrontal cortex was also observed. The medial prefrontal cortex is the core brain area of the default mode network that participates in social cognition, emotional, and self-referential processing (Gusnard et al., 2001; Amodio and Frith, 2006; Etkin et al., 2011; Wang et al., 2019a,c). The abnormal functional activities in medial prefrontal cortex have been reported in previous studies by analyses of static ALFF, local regional homogeneity, and whole-brain functional connectivity homogeneity (Yu et al., 2012; Xue et al., 2013). In addition, the middle frontal cortex and lateral orbitofrontal cortex play an important role in attention and executive control of pain-related stimuli to modulate the descending pain system (Casey, 1999; Lorenz et al., 2003; Wager et al., 2004). Thus, decreased dALFF values in the medial prefrontal cortex and middle prefrontal cortex may be related to impaired cognitive and emotion processing of pain. Moreover, we found that the changed dALFF in brain areas including the bilateral anterior insula, bilateral anterior cingulate cortex, bilateral medial prefrontal cortex, and left middle frontal cortex can well distinguish the migraine patients from controls. This finding indicates that the abnormal dynamic activities in default mode network, salience network, and executive network may be the neuropathology of migraine.

There are some limitations of our study. First, how to determine the length of sliding window is a problem for dynamic analysis. We used different window lengths to validate the findings. Second, the samples of our study are small, and the findings need to be further validated in the future studies with a larger sample.

## Conclusion

This study revealed the abnormal dynamic low-frequency oscillation in default mode network, salience network, and executive control network in migraine patients. The decreased dALFF in these areas may be associated with disrupted emotion and cognitive functions. Our findings provide new evidence that migraine could influence the brain functions leading to functional impairments of emotion and cognitions in patients.

## Data Availability Statement

The raw data supporting the conclusions of this article will be made available by the authors, without undue reservation.

## Ethics Statement

The studies involving human participants were reviewed and approved by the Third Affiliated Hospital of Chengdu Medical College, Pidu District People's Hospital. The patients/participants provided their written informed consent to participate in this study. Written informed consent was obtained from the individual(s) for the publication of any potentially identifiable images or data included in this article.

## Author Contributions

All authors have made a substantial contribution to this work.

## Conflict of Interest

The authors declare that the research was conducted in the absence of any commercial or financial relationships that could be construed as a potential conflict of interest.
